# Editorial Notes

**Published:** 1902-12

**Authors:** 


### Editorial Notes.

The **Medical Faculty of Univerity College** may be congratulated in its good fortune in having been able to secure the services of Sir Victor Horsley, F.R.S., F.R.C.S., at the annual distribution of prizes on October 24th, 1902. The address given on that occasion was described by Professor Nelson Dobson as "a happy blend of erudition, common sense, and eloquence." It must be encouraging to the present generation of medical students to learn that the number of men now entering the profession is fewer than it was ten years ago, and that the present is a most extraordinarily favourable time to enter it; this may be so, but it is very questionable whether the position of the medical man in our cities has in any way improved, for the multiplication of clubs and cheap dispensaries must make it very difficult for the average practitioner to secure an income adequate to the work he has had and has to do.

It was also pointed out that the activity of the General Medical Council in weeding out the evil-doers of the profession has never been recognised by the public, who are not commonly aware of the fact that the arrest of professional misconduct has been one of the most important functions of the Council, which has so won the gratitude of the profession in general by doing its best to keep up the social status at a high level. He gave also a few words of eulogium of what is commonly called medical etiquette, which he considered to be an outcome of a sense of responsibility of the life of the patient: he considered that the assumption of mystery was a kind of professional misconduct of which the State took no cognisance, but which the medical profession had endeavoured to combat for many years past. Sir Victor Horsley concluded his address by a eulogium of the students' clubs, and a desire that they might federate their sports. We are glad to learn that every effort is being made to unite all departments of the College in the athletic field, and that the Students' Clubs Union is already a success.

The same idea, unity, was also emphasised by Sir Victor at the annual dinner, which was held at the Royal Hotel in the evening, under the presidency of Dr. Michell Clarke. Sir Victor then stated that the Medical School should soon be able to grant its own degrees, and to this end it was important to combine all the central teaching bodies of the city. Many attempts to accomplish this have been made, and we hope that the time is not far distant when these attempts will be successful.

The Annual Meeting of the **Bristol Medico-Chirurgical Society,** to which this Journal owes its existence, affords us an opportunity of alluding to the good work done by it for a period of over twenty-five years. The recurring Presidential addresses form a series of which the Society may well be proud, and the last, which we publish in this number, is one of the most entertaining of them all.

Mr. Munro Smith was Secretary of the Society for many years, and the Presidential office is his natural due in recognition of all the good work he has done on its behalf. He was succeeded in the secretarial office by Mr. Paul Bush, C.M.G., who will also in the ensuing year be promoted to the office of President.

One of the most useful developments of the Society has been this Journal, which commenced under the editorship of Mr. Greig Smith in 1883. This number completes the twentieth volume of a work which has ministered much to the mental activity and prosperity of the medical profession of the neighbourhood.

Another good work done by the Society was the establishment of a Medical Library, and the Report of the Honorary Librarian (page 378) shows that we have an exceedingly good medical library in Bristol; this is mainly due to the persevering energy of the late librarian, Mr. L. M. Griffiths, and the work is being well maintained by his successor, Mr. C. King Rudge. The story of the genesis of this library was told in the *Journal*, vol. ix., pp. 313–6, and it seems necessary to remind the members of the Society that "the library is the direct outcome of the success of the *Journal*: if it is to increase its efficiency, it must do so to a very large extent by the members of the Society extending in every possible way the circulation of the *Journal*, and by their own contributions raising more and more the literary standard of the publication." Some further details of the growth of the Library since the year 1901 will be found on page 376.

The work of book reviewing has always been an important part of the functions of this *Journal*. These reviews are commonly written by members of the Society who have a special knowledge of the special subject, and we have always endeavoured to give judicious criticism rather than indiscriminate laudation. A writer in the *Medical Record* (Oct. 4th, 1902, p. 539) remarks that "an independent periodical seeking only the good of its readers and endeavoring to minister to their real needs must exert its beneficial influence in discriminating the good from the bad and in rendering a candid criticism of the best fruits of an open market. . . It is never a question if the volume will sell, but if it will fulfil the higher mission of profit to the reader."

We commend these remarks to the writers and the readers of our book reviews, and we trust that the portion of each of our numbers allotted to reviews will continue to be of interest and to give valuable instruction to our readers.

The resignation by **Mr. W. H. Harsant**, F.R.C.S., of the office of surgeon to the Bristol Royal Infirmary is the first of many changes in the medical and surgical staff of the Infirmary which must of necessity arise in the course of a few years.

Mr. Harsant was elected Assistant Surgeon in 1879, and continued in this office till 1885, when he was elected Surgeon; the institution owes him a great debt of gratitude for his twenty-three years of assiduous and painstaking work, and many generations of students are much indebted to him for the punctuality and attention which he has always given to his clinical duties. His friends will all wish him many years of continued activity in the less exacting work of his private and consulting practice at home.

The Executive Committee of the Gloucester, Somerset and Wilts Branch of the National Association for the Prevention of Consumption has not been as successful as we could wish in obtaining funds for the proposed Sanatorium at Winsley. Meetings have been held in various places, and a subscription list will shortly be published. At the meeting held in Gloucester the following remarks were made by Dr. Lionel Weatherly, who said:—

"It was proposed that the accommodation should at first be sixty beds, but as the patients were only retained for about three months, they would probably be able to treat at least three times that number of patients. They started the idea of building this Sanatorium solely and wholly from the philanthropic public and the self-help from the working classes. Owing possibly to the death of the Queen and the memorials to her memory got up all over the country, and this year the Coronation of the King and the consequent expenses to the public connected therewith, a check had been placed upon this philanthropic purpose, and they were bound to do what other sanatoria had done in other counties—come and ask the Town Councils to give them some support. That help could be given in this way: the Town Council could vote a sum to build one, two, or more beds in the sanatorium, and promise a further sum to maintain them. They would have the sole right of the beds they maintained, and a voice in the management. They wanted to apportion fifteen beds to each county (Bristol being reckoned as a county), and of that fifteen ten, they hoped, would be supported by the town councils, the county councils, by bodies of working men, by factories, or employers. These beds would belong to the bodies who maintained them. The other five beds for each county would be free to nominate any case by the payment of a small fee, not exceeding 10s. per week. It might be said—Would it not be better to let each county or district have its own sanatorium? His answer was that the experience in Germany, where they had spent hundreds of thousands of pounds in connection with this matter, was that it was absolutely essential that not only should the patients have fresh air and food, but they also needed hourly skilled medical supervision, and it was only under such skilled medical supervision that they could hope to arrest the disease in its early stages in the space of three or four months. Therefore, on the score of economy, it was not practicable to have an institution with less than sixty beds. Bath had unanimously voted them £500 to build and maintain two beds, and Bristol City Council had unanimously passed a resolution to build and maintain four beds."

One of the most recent researches has been carried out by the Medico-Psychological Association of Great Britain and Ireland, which has done good work in appointing a Tuberculosis Committee for the investigation and collection of evidence and for practical suggestions as to the isolation of phthisical patients in Asylums. The report of the Committee has recently been issued, and their two years of work, under the chairmanship of Dr. Lionel A. Weatherly, has been summarised in the conclusions on page 383.

